# Involvement of c-Myc in low dose radiation-induced senescence enhanced migration and invasion of unirradiated cancer cells

**DOI:** 10.18632/aging.203527

**Published:** 2021-09-22

**Authors:** Jyh-Der Leu, Chung-Yih Wang, Chia-Chien Lo, Min-Ying Lin, Chun-Yuan Chang, Wen-Chin Hung, Shi-Ting Lin, Bo-Shen Wang, Yi-Jang Lee

**Affiliations:** 1 Department of Radiation Oncology, Taipei City Hospital, Taipei 110, Taiwan; 2 Radiotherapy, Department of Medical Imaging, Cheng Hsin General Hospital, Taipei 112, Taiwan; 3 Department of Biomedical Imaging and Radiological Sciences, National Yang Ming Chiao Tung University, Taipei 112, Taiwan; 4 Cancer Progression Research Center, National Yang Ming Chiao Tung University, Taipei 112, Taiwan; 5 Rutgers Cancer Institute of New Jersey, Rutgers University, New Brunswick, NJ 08903-2681, USA; 6 Institute of Neuroscience, National Cheng Chi University, Taipei 116, Taiwan

**Keywords:** low dose radiation, c-Myc, senescence, migration and invasion, bystander effect

## Abstract

Ionizing radiation is known to cause cell apoptosis at high dose range, but little is known about the cellular response to low dose radiation. In this study, we found that conditioned medium harvested from WI-38 lung fibroblasts and H1299 lung adenocarcinoma cells exposed to 0.1Gy to 1Gy could enhance the migration and invasion of unirradiated H1299 cells in both 2D and 3D culturing circumstances. Low dose radiation did not induce apoptosis, but induced senescence in irradiated cells. We next examined the expression of immediately early genes including c-Myc and K-Ras. Although both genes could be up-regulated by low dose radiation, induction of c-Myc was more specific to low dose range (0.5Gy) at transcriptional and translational levels. Knockdown of c-Myc by shRNA could repress the senescence induced by low dose radiation. The conditioned medium of irradiated cells induced migration of unirradiated cells was also repressed by knockdown of c-Myc. The c-Myc inhibitor 10058-F4 could suppress low dose radiation induced cell senescence, and the conditioned medium harvested from irradiated cells pretreated with 10058-F4 also lost the ability to enhance the migration of unirradiated cells. The cytokine array analysis revealed that immunosuppressive monocyte chemoattractant protein-1 increased by low dose radiation could be repressed by 10058-F4. We also showed that 10058-F4 could suppress low dose radiation induced tumor progression in a xenograft tumor model. Taken together, current data suggest that -Myc is involved in low dose radiation induced cell senescence and potent bystander effect to increase the motility of unirradiated cells.

## INTRODUCTION

Radiotherapy is a routine approach to treat human cancers. However, it has been reported that ionizing radiation may promote tumor migration and invasion [1–4]. Because the primary portion of tumor should be destroyed by focused radiotherapy, it is speculated that cells surrounding the tumor may change their own biological properties by receiving off-target radiation with lower dose. Low dose radiation (LDR) is known to induce sublethal damage that may influence intracellular signaling of irradiated cells to increase the viability, gene mutation, motility and even cytokine secretion [5–7]. Prevention of the adverse effects caused by LDR would be important for improving the safety of radiotherapy.

Radiation has been reported to induce the bystander effect (RIBE) that allows the signal transmission from irradiated cells to non-irradiated cells [8, 9]. The responses of non-irradiated cells may be dependent on various factors, such as radiation doses or cancer types [10, 11]. In addition, the particulate radiation induced bystander effect mainly renders cell killing in non-irradiated cells that closely locate around the irradiated cells, while the electromagnetic radiation would promote the secretion of molecules that influence the biological behaviors of non-irradiated cells [12, 13]. Whether radiation induced bystander effects contribute to these adverse therapeutic outcomes is of interest to be investigated.

Ionizing radiation (IR) primarily induces DNA damage and subsequent signaling pathways and promotes senescence in normal cells and tumor cells [14, 15]. Stress-induced premature senescence is one of the radiation effects on both normal cells and tumor cells [16]. The expression of p53 and p16^INK4^ cell cycle regulatory molecules is involved in mediating the IR induced SIPS, although deficiency of p53 or p16^INK4^ does not completely abrogate SIPS caused by IR [17]. Several lines of evidence have shown that LDR is able to promote SIPS in stromal fibroblasts and lead to secretion of inflammatory cytokines to stimulate the proliferation of associated tumors [18–21]. However, the underlying mechanisms remains to be investigated.

In this study, we found that LDR below 1Gy could induce potent bystander effect in non-irradiated cancer cells. The LDR treated cells exhibited senescent phenotypes and increased expression of c-Myc immediately early gene. The ICM of c-Myc shRNA transduced cells reduced the level of senescence and the rate of migration in unirradiated cells. Use of a c-Myc inhibitor in LDR treated cells also suppressed the migration of unirradiated cells. Current data suggest that c-Myc is involved in LDR induced senescence and potent bystander effects.

## RESULTS

### The conditioned medium of LDR treated cells induced the invasion and migration of non-irradiated cells

**Figure 1:**
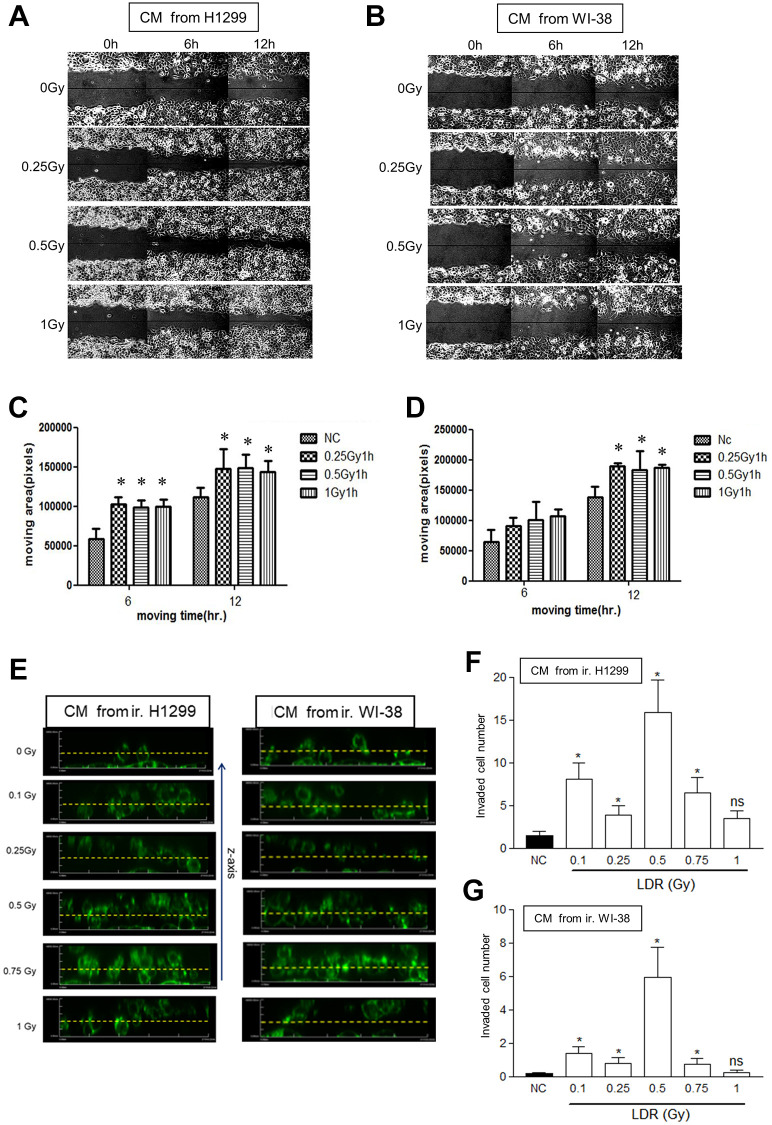
**Effects of conditioned medium (CM) from LDR irradiated cells on the migration rate of unirradiated H1299 cells.** (**A** and **B**) The wound healing assay in the monolayers using CM irradiated H1299 cells and WI-38 cells, respectively. (**C** and **D**) Measurement of the cell migration rates in the monolayers using the Image J software. (**E**) The 3D cell migration assay for analyzing the migration of H1299 cells that were treated with CM harvested from LDR treated cells. Dot line: 20 μm distance from the bottom line. (**F** and **G**) Quantification of the number of unirradiated H1299 cells migrating over 20 μm in collagen-based 3D environment in response to CM collected from irradiated H1299 cells and WI-8 cells, respectively. ^*^p < 0.05. ns: Non-significance.

At first, whether LDR irradiated cells would influence the motility of unirradiated cancer cells was investigated. WI-38 fibroblasts and H1299 lung cancer cells were exposed to X-rays below 1Gy. After 1 hour of re-incubation, the conditioned medium (CM) were harvested and added to the unirradiated H1299 cells for evaluating the migration rate using the wound healing assay. The results showed that the migration rate of unirradiated H1299 cells were increased by adding CM collected from both LDR treated cells up to 12 hours (Figure 1A and 1B). In addition, the LDR treated H1299 cells significantly enhanced the migration rate of unirradiated H1299 cells at earlier time point than WI-38 cells (Figure 1C and 1D). We also investigated the invasion of unirradiated H1299 cells following the CM treatment in the 3D environment (see Materials and Methods). Using the confocal microscopy-based cell invasion assay, we quantified cells invading upwards against gravity through the matrix; sequential z-axis sections were reconstructed and cells invading over a threshold of 20 μm were analyzed (Figure 1E). The results showed that CM of LDR irradiated WI-38 cells and H1299 cells could differently enhance the invasion of unirradiated H1299 cells in 3D culture (Figure 1F and 1G). Interestingly, it appeared that 0.5Gy LDR induced most apparent invasive ability on unirradiated H1299 cells. We also used the *in vitro* invasion assay to demonstrate that H1299 cells or WI-38 cells irradiated with 0.1Gy to 1Gy, the resultant CM could enhance the cell invasion of unirradiated H1299 cells through matrigel coated transwells within a dose-dependent manner (Supplementary Figure 1).

### Induction of senescent phenotype in cells by exposure to LDR

**Figure 2:**
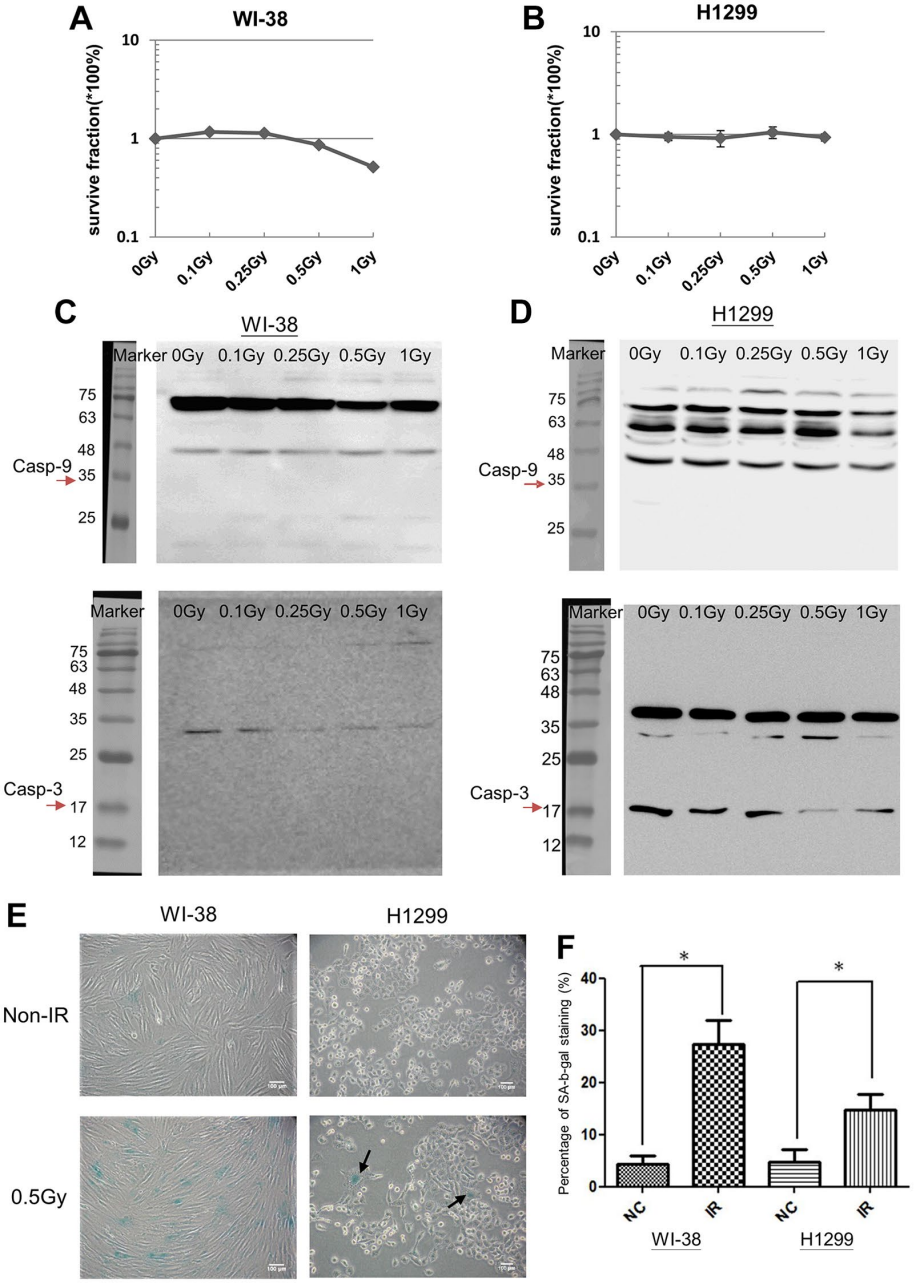
**LDR induced cell senescence but not apoptosis.** (**A** and **B**) MTT assay for analyzing the cell viability of LDR treated WI-38 cells and H1299 cells, respectively. (**C** and **D**) Western blot analysis of Caspase-3 and Caspase-9 for detecting the cleaved proteins in LDR treated WI-38 cells and H1299 cells, respectively. (**E**) SA-β-gal analysis for determining the level of cell senescence induced by LDR. (**F**) Quantification of X-gal stained cells before and after LDR. ^*^p < 0.05.

To determine whether LDR would influence cell viability, the colony formation assay was used to measure the survival fractions of irradiated WI-38 cells and H1299 cells. The results showed that apparent reduction of survival fraction was not detected until WI-38 cells were exposed to 1Gy (Figure 2A). On the contrary, the survival fractions of H1299 cells did not decrease between 0.1Gy to 1Gy (Figure 2B). The radiosensitivity of H1299 cells was also not influenced by pre-exposure of 0.5Gy followed by high dose ranged from 2Gy to 8Gy (Supplementary Figure 2). The degradations of apoptotic initiator caspase-9 and terminator caspase-3 were not detected in both cell types after LDR irradiation (Figure 2C and 2D). Interestingly, LDR could induce the expression of senescence associated β-galactosidase (SA-β-gal) in both WI-38 cells and H1299 cells (Figure 2E), The SA-β-gal staining before and after LDR was quantified in both cell types (Figure 2F). These results suggested that LDR exposure would induce cell senescence.

### Effects of LDR on immediately early genes

**Figure 3:**
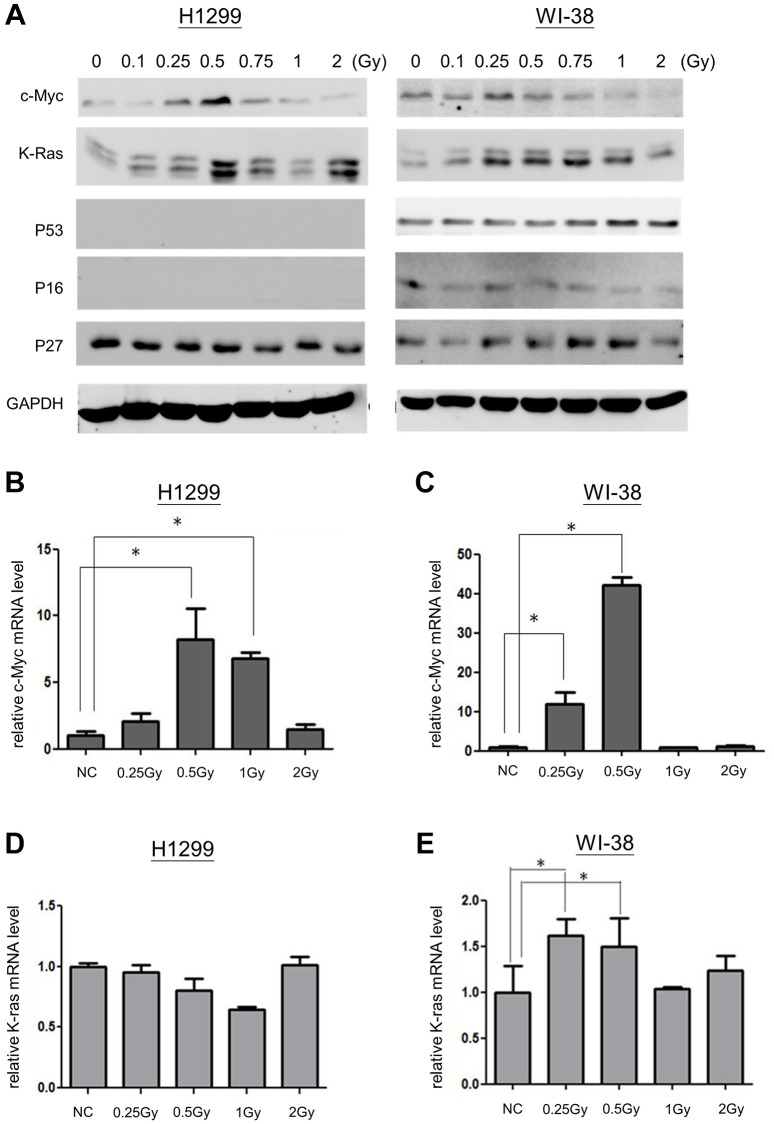
**Expression of c-Myc and K-Ras in LDR induced senescence.** (**A**) Western blot analysis for detecting the expression of c-Myc, K-Ras, p53, p16 and p27 in cells exposed to LDR. (**B** and **C**) qPCR was used for detection of c-Myc mRNA levels in H1299 cells and WI-38 cells after ionizing radiation, respectively. (**D** and **E**) The expression of K-Ras mRNA in H1299 cells and WI-38 cells exposed to ionizing radiation, respectively. ^*^p < 0.05.

LDR belongs to low lethal irradiation that may induce immediately early genes, so called proto-oncogenes [22]. Up-regulation of oncogene-induced senescence (OIS) has been extensively reported [23, 24]. Here we investigated whether LDR could influence the expression of c-Myc and K-Ras proto-oncogenes for LDR induced senescence. Compared to K-Ras, c-Myc of both WI-38 cells and H1299 cells exhibited a narrow spectrum induced by LDR (between 0.25Gy and 0.5Gy) (Figure 3A). Quantitative PCR (q-PCR) also showed that the mRNA level of c-Myc was also induced by LDR in both cell types, and it appeared that 0.5Gy could induce maximum c-Myc mRNA expression at 1 hour after exposure (Figure 3B and 3C). Notably, the c-Myc mRNA was also induced in H1299 cells but not WI-38 cells after they were exposed to 1Gy, but the c-Myc protein levels were not induced in both cell types. For K-Ras, the mRNA level of WI-38 cells but not that of H1299 cells was induced by LDR up to 0.5Gy (Figure 3D and 3E), suggesting that the expression of K-Ras was mainly up-regulated by post-translational pathway. As c-Myc was only induced by LDR lower than 1Gy, we further examined whether c-Myc is important for LDR induced senescence.

### Association of c-Myc with LDR induced bystander effect

**Figure 4:**
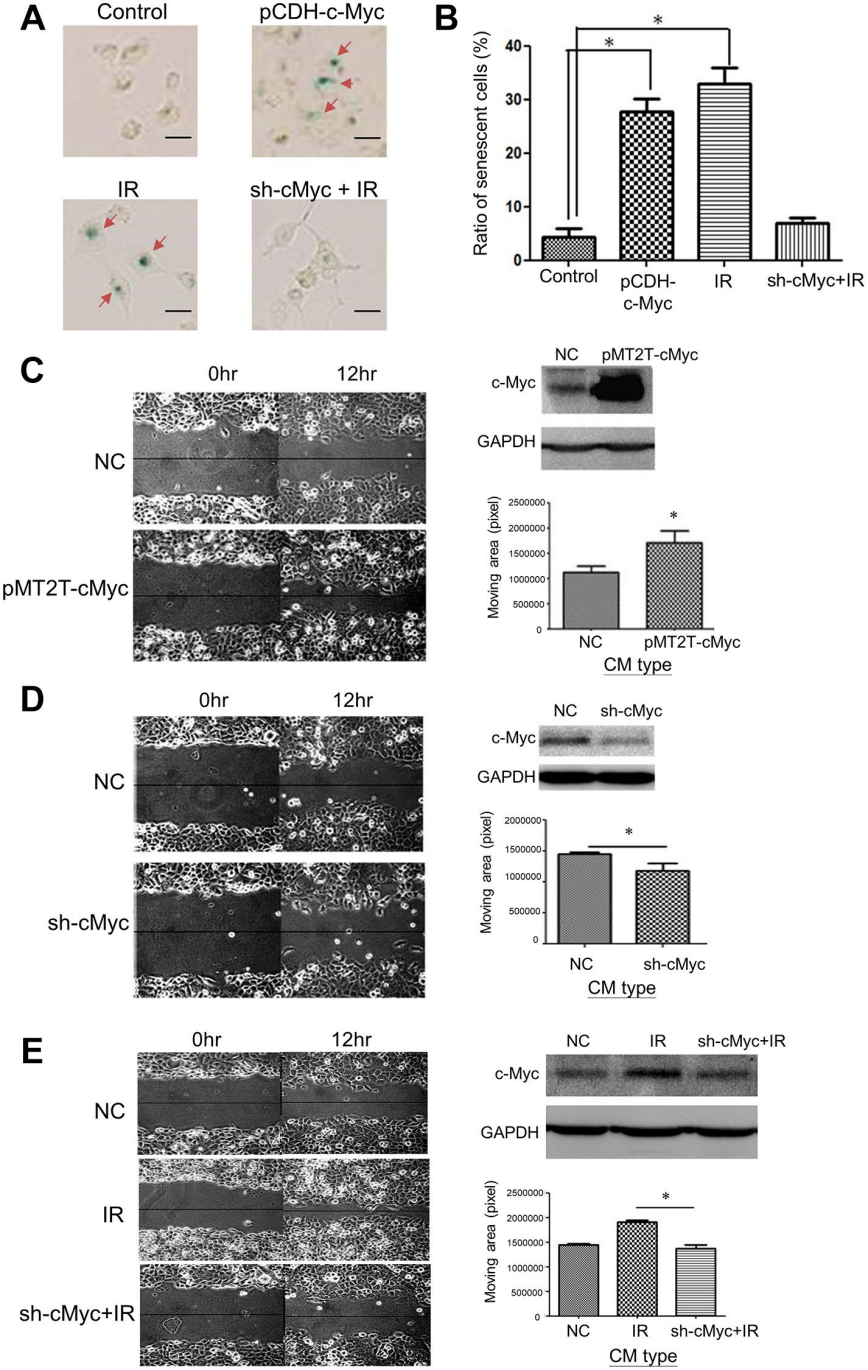
**Effects of c-Myc expression on LDR induced senescence and unirradiated cell migration.** (**A**) The level of SA-β-gal staining was increased by over-expression of c-Myc in H1299 cells. Radiation induced SA-β-gal was reduced by knockdown of c-Myc using shRNA. Scale bar: 100 μm. (**B**) Quantification of cell number stained by X-gal after different treatments. (**C**) Comparison of cell migration rate between normal medium and CM collected from c-Myc cover-expressing cells. (**D**) Comparison of cell migration rate between normal medium and CM collected from c-Myc knockdown cells. (**E**) Enhanced migration of H1299 cells by CM collected from LDR irradiated cells was suppressed by knockdown of c-Myc in irradiated cells. ^*^p < 0.05.

Next, we investigated if the condition medium (CM) of LDR enhanced migration in unirradiated cells is associated with the expression of c-Myc. Suppression of c-Myc mRNA expression using siRNA could compromise the LDR induced senescence. We have shown that LDR could induce the expression of SA-β-gal in H1299 cells, while knockdown of c-Myc would suppress this effect (Figure 4A). Over-expression of c-Myc in H1299 cells also exhibited similar effects with LDR on induction of SA-β-gal (Figure 4A and 4B). The CM of c-Myc over-expressing cells and c-Myc silencing cells could enhance and reduce the migration of normal H1299 cells as demonstrated by the wound healing assay, respectively (Figure 4C and 4D). Compared to LDR treated cells, the CM of knockdown of c-Myc in LDR treated cells did not enhance the migration rate of unirradiated cells (Figure 4E). These results suggest that c-Myc is associated with LDR mediated bystander effects on increase of cell migration.

### Inhibition of LDR caused bystander effects using c-Myc inhibitors

**Figure 5:**
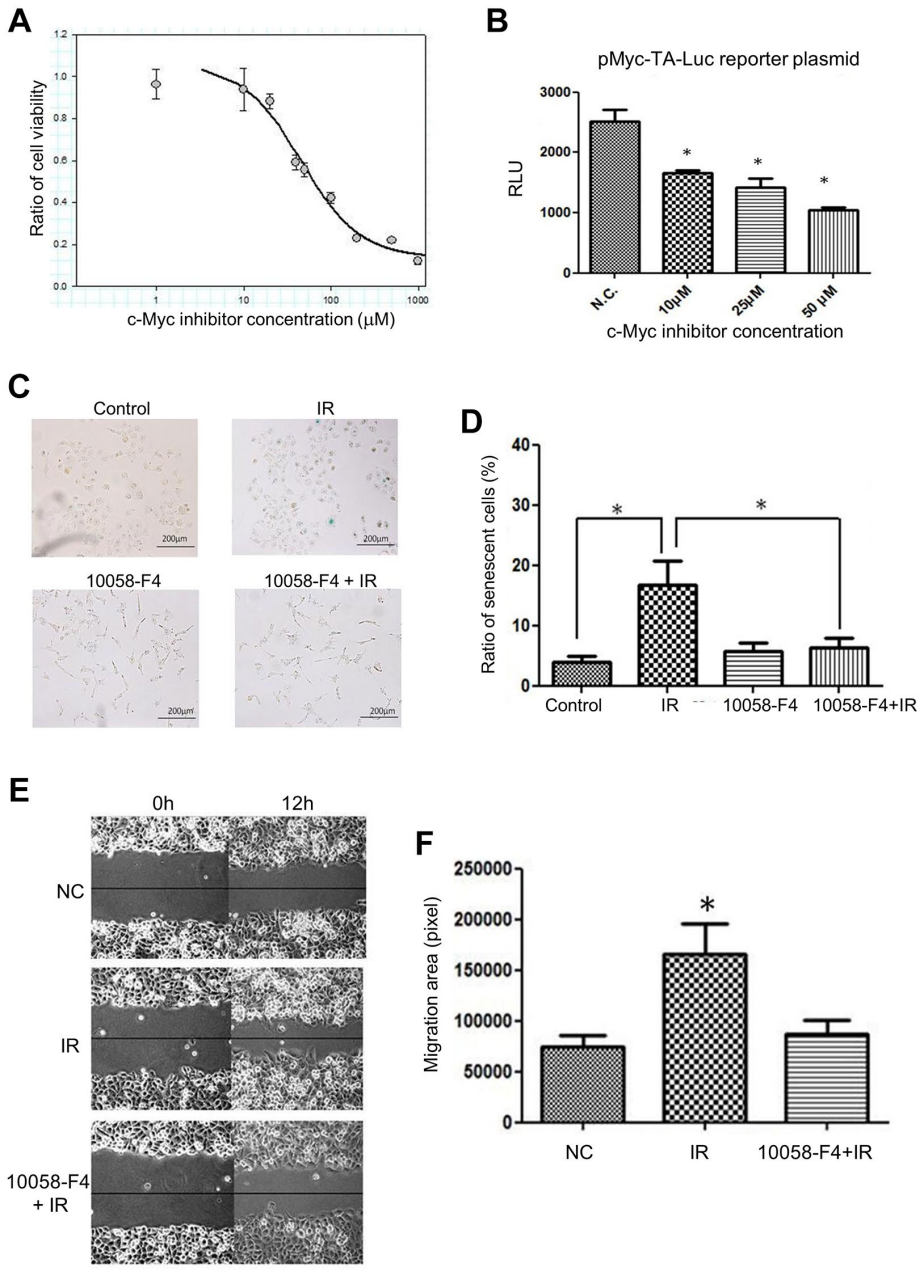
**Effects of c-Myc inhibitor (10058-F4) on LDR induced senescence and unirradiated cell migration.** (**A**) Cell survival curve of H1299 cells treated with increased dose of 10058-F4. (**B**) Luciferase reporter gene assay for demonstrating the effects of 10058-F4 on suppressing c-Myc transcriptional activity in H1299 cells. (**C**) 10058-F4 suppressed IR (0.5Gy) induced senescence. (**D**) Quantification of SA-β-gal staining in cells exposed to IR or 10058-F4. (**E**) Comparison of the effects of CM collected from cells exposed to IR with or without the presence of 10058-F4 (50 μM) on the migration rates of unirradiated cells. (**F**) Quantification of cell migration by wound healing assay. ^*^p < 0.05.

To further investigate whether inhibition of c-Myc activity is important for compromising the LDR induced bystander effects, a clinical c-Myc inhibitor (10058-F4) was used to treat cells before irradiation. The colorimetric cytotoxicity assay showed that the IC50 of 10058-F4 was 45.01 μM for H1299 cells (Figure 5A). The luciferase reporter gene assay showed that the c-Myc transactivation activity of H1299 cells was suppressed by 10058-F4 from 10 μM to 50 μM (Figure 5B). Additionally, the LDR induced cell senescence was suppressed by 10058-F4, which did not influence the cell senescence (Figure 5C and 5D). Moreover, the pretreatment of 10058-F4 could suppress the CM of LDR promoted cell migration (Figure 5E and 5F). These results further support the role of c-Myc involved in LDR mediated bystander effect.

### Analysis of cytokines in LDR treated cells

**Figure 6:**
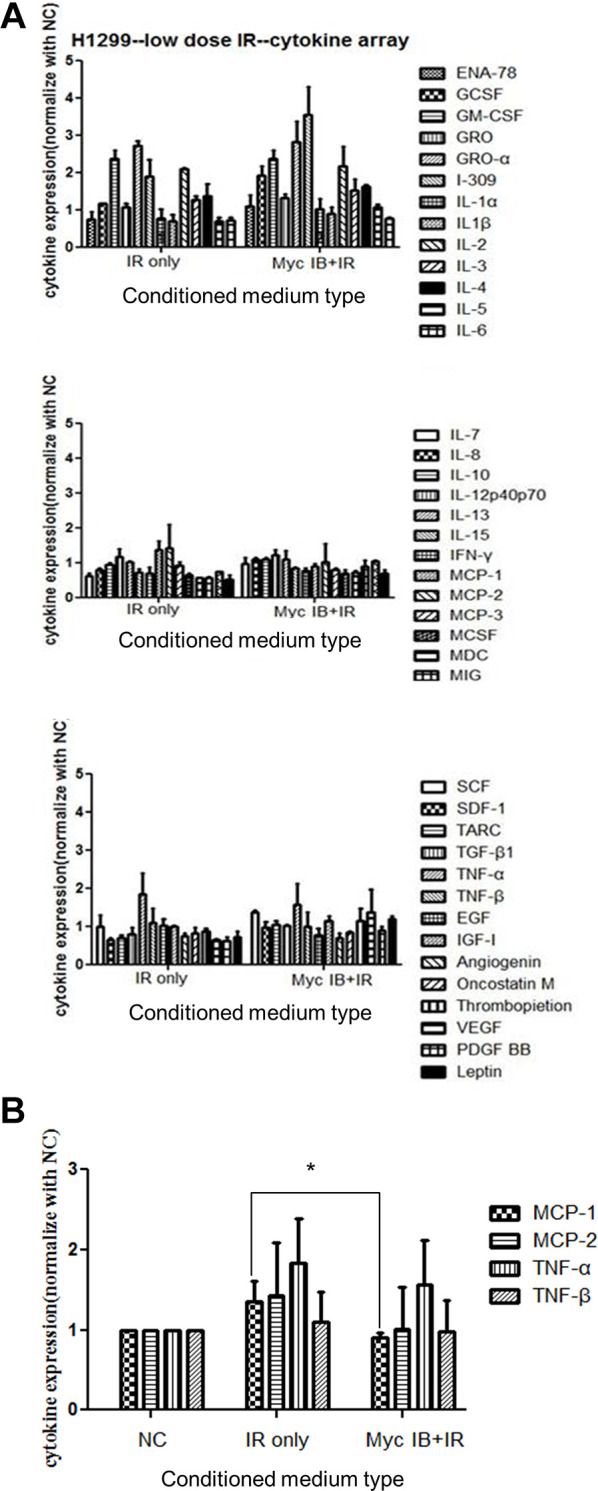
**Cytokine array analysis of CM from cells treated with LDR with or without the presence of c-Myc inhibitor.** (**A**) The cytokine array was used to detect the cytokines released in CM of LDR (0.5Gy) treated H1299 cells with or without c-Myc inhibitor (Myc IB, 50 μM). Densitometric quantification of 42 cytokines detected in the cytokine array. Data represent the means of duplicated dots. (**B**) Effects of CM from cells treated with LDR with or without the presence of c-Myc inhibitor on the expression of MCP1, MCP-2, TNF-α and TNF-β. ^*^p < 0.05.

The LDR induced change of cytokines released to culture medium was subsequently examined using a human cytokine array that contains 42 different cytokines (Supplementary Figure 3A). The culture media were collected from untreated cells, X-rays irradiated cells, or irradiated cells pretreated with 10058-F4, and they were concentrated and run on an SDS-PAGE (Supplementary Figure 3B). After addition of these concentrated culture media to the array, the blots were analyzed by the Image J software to quantify the intensity of each dot. The expression of all cytokines was compared between cultured medium of LDR with or without the treatment of 10058-F4 (Figure 6A). It appeared that only MCP-1, MCP-2, TNF-α and TNF-β were differently up-regulated by LDR compared to untreated cells. Interestingly, MCP-1 was the only cytokine that could be suppressed by 10058-F4 (Figure 6B). Radiation induced MCP-2 also slightly suppressed by c-Myc inhibitor. On the other hand, several cytokines such as I-309, leptin, and VEGF tend to be up-regulated by combining treatment of c-Myc inhibitor and radiation (Supplementary Table 1).

### c-Myc inhibitor suppresses LDR induced tumor growth in the xenograft tumor model

**Figure 7:**
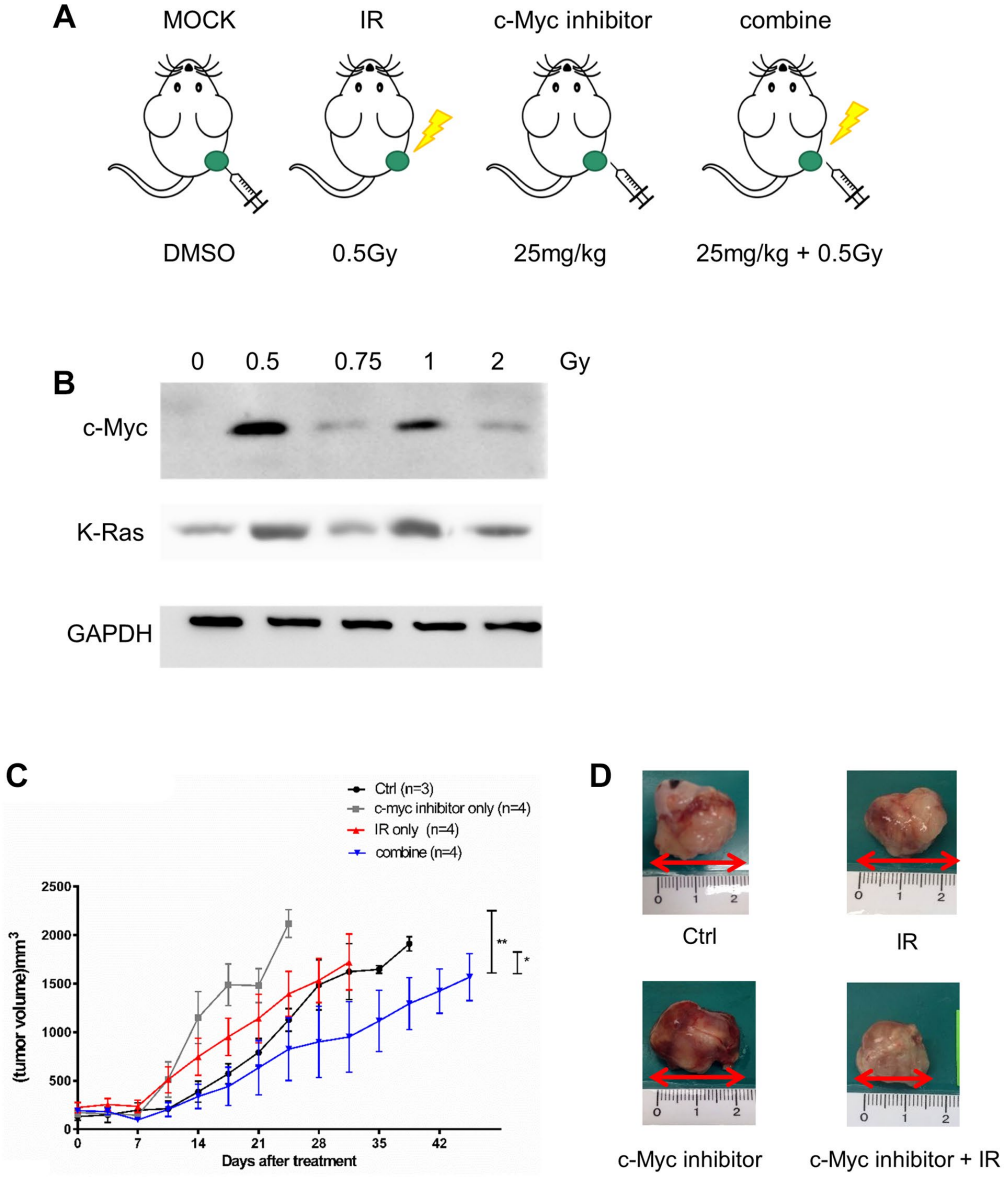
**Effects of c-Myc inhibitor on LDR irradiated NSCLC xenograft tumor.** (**A**) Design of xenograft tumor model for treatment of LDR and c-Myc inhibitor. (**B**) Detection of c-Myc and K-Ras protein expression in LDR irradiated tumors formed by H1299 cells. (**C**) Measurement of tumor growth by caliper after LDR irradiation (0.5Gy) with or without the treatment of 10058-F4 (25 mg/kg). (**D**) Comparison of tumor sizes excised from tumor-bearing mice with different treatment for 3 weeks. ^*^p < 0.05.

We next designed a xenograft tumor model to investigate the effect of LDR combining c-Myc inhibitor on tumor progression *in vivo* (Figure 7A). LDR could induce the highest expression of c-Myc in H1299 cells formed xenograft tumor at 0.5Gy, while K-Ras could be induced to similar levels at 0.5Gy and 1Gy (Figure 7B). The tumor growth curves showed that the tumor growth rates were increased by LDR and c-Myc inhibitor treatment alone, but a combination of c-Myc and LDR reduced the tumor growth over 1 month (Figure 7C). The size of excised tumors of tumor-bearing mice with the combined treatments were smaller than that of control and single treatment of LDR and c-Myc inhibitor (Figure 7D). As we did not detect the metastasis of H1299 cells in the xenograft tumor model, we also designed a synergistic tumor model using the murine triple-negative 4T1 breast cancer cells. The migration rate of 4T1 cells was also increased after they were exposed to LDR (Supplementary Figure 4A). Additionally, the metastasis of LDR irradiated 4T1 tumors was enhanced compared to the untreated control, while i.p. injection of c-Myc inhibitor right after irradiation could suppress the metastasis of 4T1 tumors (Supplementary Figure 4B). Current data suggest that inhibition of c-Myc would also compromise the LDR induced tumor progression *in vivo*.

## DISCUSSION

Radiotherapy has been reported to induce metastasis or a pro-metastatic effect of local controlled cancers [25–27]. Fractionation radiotherapy using 2Gy per fraction is commonly applied for local controlling the tumor lesion and reducing the damage of normal tissues [28]. In contrast, when the dose is <1Gy, so called LDR, angiogenesis, immunological responses, and accelerated senescence can be induced in tumor-associated host endothelial cells or stromal fibroblasts to establish a microenvironment for tumor metastasis or progression [7, 18, 19, 27, 29]. In agreement with previous reports, our data demonstrated that the ICM collected from LDR treated cells could promote the invasion and migration of unirradiated cancer cells. Although the tumor-associated host cells are regarded the targets of LDR and are involved in promoting malignancy, we found that medium from irradiated cancer cells per se could also increase the invasion and migration rates of non-irradiated cancer cells. For mammalian breast cancer cells, high dose radiation has been reported to promote the invasion of non-irradiated cancer cells [2, 30]. Out results suggest that LDR could also stimulate cancer cells and initiate potent bystander effect.

Radiation induced DNA damage would lead to apoptosis and senescence, but LDR induced lower level of DNA damage favors cell senescence but not apoptosis in normal cells [31–33]. Although the level of radiation-induced premature senescence is increased in higher radiation dose [31], our data showed that radiation dose below 1Gy could also induce cell senescence. As we have detected the expression of c-Myc oncogene just 1 hour after LDR, the association of this immediately early gene with LDR induced senescence may be important for the potent bystander effect. LDR has been reported to induce apparent "immediately early response" genes in human lymphoblastoid cells after they were exposed 0.5Gy for 1 hour [22]. We showed that a similar phenomenon could be detected in WI-38 cells and H1299 cells after irradiation. In H1299 cancer cells, however, they are deficient in both p53 and p16^INK4^ expression. LDR induced senescence in this cell type may be not dependent on tumor suppressive mechanisms. Since knockdown of c-Myc expression by siRNA could suppress the senescent phenotype in LDR treated cells, it suggests that c-Myc would mediate LDR induced senescence in cells lacking p53 and p16^INK4^. Importantly, over-expression and knockdown of c-Myc expression could also increase and decrease the migration rate of untransfected cells by their condition medium, respectively. These results further support that increase of c-Myc would promote senescence to promote the migration of other cells.

According to current data, both c-Myc and Ras could be induced by low dose radiation at 0.5Gy in H1299 cells. However, Ras was also induced at higher dose up to 2Gy compared to c-Myc. We did not overlook the role of Ras in mediating low dose radiation induced cell senescence, but we focused on c-Myc because it was induced by a more specific low dose than Ras. Indeed, we have found that knockdown of Ras using Ras shRNA could reduce the ratio of senescence in H1299 cells exposed to 0.5Gy (Supplementary Figure 5). Because c-Myc and Ras triggered different pathways to act as oncogenes but they also collaborate to promoter cancer progression [34]. It would be interesting to investigate whether inhibition of both oncogenes would synergistically inhibit low dose radiation induced senescence and related bystander effects in the future.

Over-expression of oncogenes can increase cellular ROS and lead to inappropriate DNA synthesis and DNA damage, which is known as oncogene-induced senescence [35]. Ras is the first oncogene defined to induce senescence depending on the activation of p53 and p16^INK4^ [36]. Several types of oncogenes have been reported to induce senescence in normal fibroblasts or benign tumors after over-expression, including Raf, cyclin E, mos, cdc6, E2F, Akt [37–41]. Interestingly, c-Myc oncogene has also been reported to induce senescence when Werner gene (*WRN*) or cyclin- dependent kinase 2 (CDK2) is absent [42, 43]. Although LDR can induce the expression of c-Myc and Ras, it remains unclear whether c-Myc and Ras are involved in LDR induced senescence. Our current data showed that knockdown or c-Myc or using c-Myc inhibitor could suppress the LDR induced senescence, suggesting that LDR may trigger oncogene-induced senescence through c-Myc. On the other hand, because LDR induced Ras expression is only detected in WI-38 cells but not H1299 cells, the role of Ras in LDR induced senescence remains to be elucidated.

The c-Myc inhibitor (10058-F4) has been developed to inhibit the interaction and function of c-Myc and its associated protein Max, and inhibit the tumor growth *in vivo* [44, 45]. However, it is unclear whether c-Myc inhibitor provides therapeutic benefits for radiotherapy. We have shown that 10058-F4 inhibited LDR-induced senescence and the conditioned medium enhanced migration of unirradiated cells (Figure 5). Based on the concept of senescence associated secretion phenotype (SASP), we used cytokine-based protein array to detect the possible changes of cytokines secreted to conditioned medium after LDR with or without addition of c-Myc inhibitor. The current data showed that MCP-1 could be up-regulated in conditioned medium by LDR, and the level was significantly decreased by adding c-Myc inhibitor. Although TNF-α was also increased, its expression was not suppressed by c-Myc inhibitor. MCP-1 and c-Myc belong to slow-kinetics subset of immediately early genes that are expressed at maximum level after 60 minutes of stimulations [46, 47]. Accumulated literatures have suggested that MCP-1 is associated with promotion of cancer progression by recruiting immunosuppressive macrophage or inducing angiogenesis at tumor site [48–52]. Whether MCP-1 is also involved in LDR induced c-Myc and senescence associated secretory particles is of interest to be investigated. Nevertheless, we do not exclude other cytokines that may be also induced or reduced by LDR because only forty-four cytokines were analyzed in this study. We have mentioned that I-309, leptin, and VEGF were up-regulated in conditioned medium of H1299 cells exposed to LDR combining c-Myc inhibitor in the result section. The granulocyte colony-stimulating factor (G-CSF) was also induced to a similar level, that is, over 1.5-fold compared to LDR alone. G-CSF can induce the migration of hematopoietic stem cells CD34+ cells [53]. I-309 can promote the migration of human monocytes [54]. VEGF is well known to promote the migration of endothelial cells, and leptin and is reported to induce the migration of cancer cells [55, 56]. The role of these up-regulated cytokines on the suppression of migration of unirradiated H1299 cells remained to be investigated.

We set the end-point of tumor size at 2000 mm^3^ by following the regulation of IACUC of our institute. At this condition, we could not detect the metastasis of H1299 cells *in vivo*. A previous report has demonstrated that metastasis of H1299 xenograft tumor would be detected around 2800 mm^3^ [57]. Although we did not detect tumor metastasis, we still could detect the benefit of combined treatment of IR and c-Myc inhibitor on reduction of tumor growth. It is believed that the tumor metastasis should be suppressed or delayed by this treatment. In the Supplementary Figure 4, we have tried to examine the combined effects of IR and c-Myc inhibitor on reduction of metastasis using the 4T1 synergistic tumor model. As 4T1 cells are known as triple-negative murine breast cancer cells with high metastatic ability [58], current result may provide an evidence that c-Myc inhibitor would suppress low dose radiation induced tumor migration in different cancer types.

In summary, current data showed that cells exposed to LDR would induce bystander effect that promotes migration and invasion of non-irradiated tumor cells. As knockdown of c-Myc or use of c-Myc inhibitor suppressed LDR induced senescence in cultured cells as well as the conditioned medium enhanced migration of unirradiated cells, it is interesting to further investigate whether inhibition of c-Myc is important for suppressing potent bystander effects during radiotherapy.

## MATERIALS AND METHODS

### Cell cultures

Human non-small lung cancer H1299 cells and T-antigen transformed human embryonic kidney 293T cells and cultured in Dulbecco’s modified Eagle’s medium (DMEM). Human diploid WI-38 fibroblasts were cultured in minimum essential media (MEM). These cell lines were obtained from American Type of Culture Collection (ATCC). Murine 4T1 breast cancer cell line was a generous gift of Dr. Yueh-Hsin Ou in National Yang-Ming University, and was cultured in RPMI1640 medium (GIBCO^®^ Invitrogen Inc., Carlsbad, CA, USA). All media were supplemented with 10% fetal bovine serum (FBS, HyClone^®^ Thermo, Waltham, MA, USA), 2 mM L-glutamate, 50 unit/ml penicillin and 50 μg/ml streptomycin (Invitrogen, Carlsbad, CA, USA). Cells were maintained in a 37°C, humidified incubator (5% CO_2_ and 95% air) and passaged every two days. However, WI-38 cells were only cultured up to two weeks because of spontaneous senescence.

### Plasmids

The pMyc-TA-Luc reporter plasmid is a gift from Dr. Lih-Yuan Lin in National Tsing-Hua University, Hsinchu, Taiwan. This plasmid contains a luciferase gene fused to six tandem repeats in the promoter region. The pCDH-cMyc plasmid is a lentiviral based c-Myc gene expressive construct (Addgene, Cambridge, MA 02139 USA). The pMT2T-Myc plasmid kindly provided by Dr. Muh-Hwa Yang from Yang-Ming University, Taipei, Taiwan. The pCMV-ΔR8.91, pMD.G, and pLKO.1-cMyc shRNA plasmids were obtained from RNAi Core Facility of Academia Sinica, Taipei, Taiwan.

### Reagents

(Z, E)-5-(4-Ethylbenzylidine)-2-thioxothiazolidin-4-one, also named 10058-F4, is a c-Myc inhibitor [59]. A 50mM stock solution in dimethyl sulfoxide (DMSO) was made and kept at 4°C.

### Radiation source

Cells were irradiated using the cabinet RS 2000 Biological Irradiator (Rad Source Technologies, Inc., Suwanee, GA, USA).The dose rate of irradiation is 17.7mGy/sec.

### Wound healing assay

Exponentially growing H1299 cells and WI-38 cells were irradiated at different doses as indicated. The media were collected one hour after exposure and added ton confluent H1299 and WI-38 cell cultures, respectively, which were scratched straightly at three separate positions using a yellow pipette tip and marked outside the plate. The healing rates of incised wounds were visualized and captured under a light microscope at 6 and 12 h. After 24 hours of transfection, the medium was replaced with normal culture medium for another 24 hours, and this conditioned medium was harvested and added to untransfected cell culture for wound healing assay.

### Three-dimensional (3D) cell migration assay

This assay was performed according to a previous report with slightly modifications [60]. In brief, cells were cultured in normal medium overnight. The medium was then replaced with fibrillary bovine dermal collagen solution that mixed in CM collected from irradiated cells (1.7 mg/mL in medium). The plates were incubated in the humidified incubator for another 24 h. Subsequently, the plates were fixed with 4% paraformaldehyde and stained with Alexa-488 conjugated phalloidin. The plates were visualized using a laser confocal microscope, and the confocal Z sections were collected at 50 μm from the bottom of plates. To obtain the 3D migration imaging of cells, the collected sequential Z sections were reconstructed using the Olympus FV10-ASW 1.7 software. Cells migrating over 20 μm were counted, and three random areas of the reconstructed imaging were analyzed.

### *In vitro* invasion assay

Matrigel (BD Biosciences, San Jose, CA, USA) was mixed with 25 μl serum-free medium (1:3) and added into transwells (24 Well Millicell 8.0 mm; Millipore Co., Billerica, MA, USA) that can be fit into the 24-well plate. After the matrigel was solidified, H1299 cells (1 × 10^4^) were mixed with 200 μl serum-free medium and added into each transwell. The transwells were separately placed onto the 24-well plate that had been filled with medium harvested from cells treated with various doses of radiation. After 24 hours of incubation, each transwell was cleaned using the cotton stab and washed with 1 × phosphate-buffered saline (PBS), and then stained with 1.25% crystal violet solution in ethanol for 2 min. The transwells were rinsed and the membranes were cut and placed onto slides for microscopic visualization (Leica DM IRB, Wetzlar, Germany).

### Colony formation assay

Exponential growing cells were trypsinized, counted and resuspended in T25 flasks for X-ray exposure (0.1Gy to 1Gy). Fifty or one hundred irradiated cells were then seeded in 6 cm dishes and incubated for 14 days without disturbance. Formed colonies were stained with 0.02% crystal violet solution (w/v in 75% ethanol). The plating efficiency was determined as the ratio of the number of colonies divided by the number of cells seeded. The surviving fraction was determined by the ratio of plating efficiencies of irradiated cells compared to unirradiated control. Each datum represents the mean of three independent experiments ± S.D.

### Senescence-Associated β-galactosidase (SA-β-gal) staining

The senescent levels of H1299 and WI-38 were determined using the SA-β-gal staining. Briefly, irradiated cells were rinsed with phosphate buffered saline, and then fixed with 2% formaldehyde and 0.2% glutaraldehyde for 5 min at room temperature. The fixed cells were incubated with X-gal staining solution (40 mM citric acid/sodium phosphate (pH = 6.0), 1 mg/mL X-gal, 5 mM potassium ferricyanide^2+^, 5 mM potassium ferricyanide^3+^, 150 mM NaCl, 2 mM MgCl_2_ in distilled H_2_O) at 37°C for 16 h after PBS. Stained cells were visualized under the light microscope and counted.

### Western blot analysis and antibodies

Protein lysate was extracted from cells using protein lysis buffer (50 mM Tris-HCl, 120 mM NaCl, 0.5% NP-40) with 2% PMSF and quantified using the Bio-Rad Protein Assay (Bio-Rad Laboratories Inc., Hercules, CA, USA). Lysate was mixed with sampling buffer (250 mM Tris-HCl (pH 6.8), 10% sodium dodecyl sulfate (SDS), 30% glycerol, 5% β-mercaptoethanol, 0.02% bromophenol blue), denatured by boiling, and run on SDS-polyacrylamide gel electrophoresis. Gel was then electrotransferred to a nitrocellulose membrane (BioTraceTM NT; Pall, Port Washington, NY, USA), and the membrane was blocked in 4% skim milk (150 mM NaCl, 10 mM Tris-HCl (pH 8.0), and 0.1%Tween-20) for 2 h. The membrane was then incubated with primary antibody overnight followed by horseradish peroxidase (HRP)-conjugated secondary antibody. The membrane was rinsed with Western lightning plus-ECL (Perkin-Elmer Inc., Waltham, MA, USA) and the chemoluminescent signals were detected using the LAS-4000 gel imaging system (GE Healthcare Inc., Wauwatosa, WI, USA). The primary antibodies used in this study included anti-c-Myc, anti-K-RAS, anti-p53, anti-p27^Cip1^ (Millipore Co., Billerica, MA, USA), and anti-p16^INK4a^ (Santa Cruz Inc., Santa Cruz, CA, USA). Anti-glyceraldehyde 3-phosphate dehydrogenase antibody (anti-GAPDH, GeneTex Inc., Irvine, CA, USA) was used as a control. The secondary antibodies used in this study included anti-mouse and anti-rabbit antibodies (Millipore Co., Billerica, MA, USA).

### Lentivirus mediated gene delivery

The 293T cell line was seeded in a 60-mm dish, and then co-transfected with plasmid mixtures (0.25 μg pMD.G, 2.25 μg pCMV-ΔR8.91, and 2.5 μg pLKO.1-cMyc or pCDH-cMyc) for 16 hr. The culture medium was replaced with fresh medium containing 10 mg/mL bovine serum albumin. Subsequently, the medium was collected twice after 12 and 24 hours, and centrifuged at 110,000 × g for 2 h at 4°C. The pellet was resuspended in serum-free medium, mixed with 8 μg/ml of polybrene (Sigma-Aldrich Inc., St. Louis, MO, USA), and added to target cells for 24 hours of infection. Cells were then replaced with normal medium for another 24 hours and then analyzed.

### Luciferase reporter gene assay

Cells were seeded in 24-well plates and transfected with pMyc-TA-Luc plasmid using JetPEI Reagent (POLYPLUS-TRANSFECTION, Illkirch, France). After 48 hours of transfection, cells were lysed with 5X lysis buffer (Promega Co., Madison, MI, USA), which was diluted using the luciferase assay buffer (25 mM Tris/phosphate, 20 mM MgSO_4_, 4 mM EDTA, 2 mM adenosine triphosphate, 1 mM dithiothreitol). The lysate was centrifuged and the supernatant was mixed with luciferase assay buffer containing 50 mM D-luciferin (Promega Co., Madison, MI, USA). The mixture was immediately measured using the Wallac-1420 VICTOR^2^ multilabel reader (PerkinElmer Co., Waltham, MA, USA).

### Human cytokine array analysis

Cells were irradiated or combined with different treatments, and the media were collected and concentrated using Vivaspin-20 sample concentrator (GE Healthcare Bio-Sciences AB, Uppsala, Sweden). 40 μL aliquots from separately collected original medium and from final concentrated medium were separated on an 8% SDS-PAGE gel and stained with Coomassie Blue to determine the efficiency of sample concentration. Subsequently, we used the human cytokine array C3 kit (Raybiotech Inc., Norcross, GA, USA) to measure cytokine concentrations in the samples following the manufacturer’s instructions. The results were normalized to untreated control and quantified using Image J software.

### Animal tumor model

Six weeks old male nonobese diabetic/severe combined immunodeficiency (NOD/SCID) mice were anesthetized by intra-peritoneal injection of a mixture of ketamine chloride (50 mg/kg) and xylazine (15 mg/kg). Subsequently, H1299 (5 × 10^6^) cells re-suspended in 100 μl medium were subcutaneously injected in both thighs of those NOD/SCID mice using 27-gauge insulin syringes. Tumor volume was caliperly measured and calculated using this formula: length × (width)^2^/2. For the synergistic tumor model, 4T1 murine breast carcinomas were transduced with luciferase reporter gene in advanced so that the tumor growth and metastasis can be tracked using the bioluminescent imaging [61]. The luciferase gene harboring 4T1 cells (1 × 10^6^), so called 4T1-3R cells were collected and subcutaneously implanted to Balb/C mice to form syngeneic tumors. Imaging of tumor growth and development under different experimental conditions were investigated using *In vivo* Imaging System 50 (IVIS-50 Xenogen Co., Alameda, CA, USA). In brief, mice were intraperitoneal injected with 150 mg/kg D-luciferin (Caliper Co., Hopkinton, MA, USA), and anesthetized using 2–4% isoflurane in IVIS-50 system for imaging acquisition. Animal protocols have been reviewed and approved by the Institutional Animal Care and Use Committee (IACUC) of National Yang-Ming University.

### Statistical analysis

Experimental data were represented as the mean of three independent experiments ± S.D. Data were analyzed with *t*-test. The two-way analysis of variance (ANOVA) was performed for analyzing the tumor growth after treatments. *P* < 0.05 indicating statistical significance. The prism v5.0 software (GraphPad Software, Inc., La Jolla, CA, USA) was used to illustrate the quantification data.

## Supplementary Materials

Supplementary Figures

Supplementary Table 1
