# Supplementary material for: Involvement of c-Myc in low dose radiation-induced senescence enhanced migration and invasion of unirradiated cancer cells
Source: Aging (Albany NY). 2021 Sep 22;13(18):22208–31. doi: 10.18632/aging.203527 (PMC8507273; doi:10.18632/aging.203527)
Supplement: Supplementary Figures [file aging-13-203527-s001.pdf]

SUPPLEMENTARY FIGURES

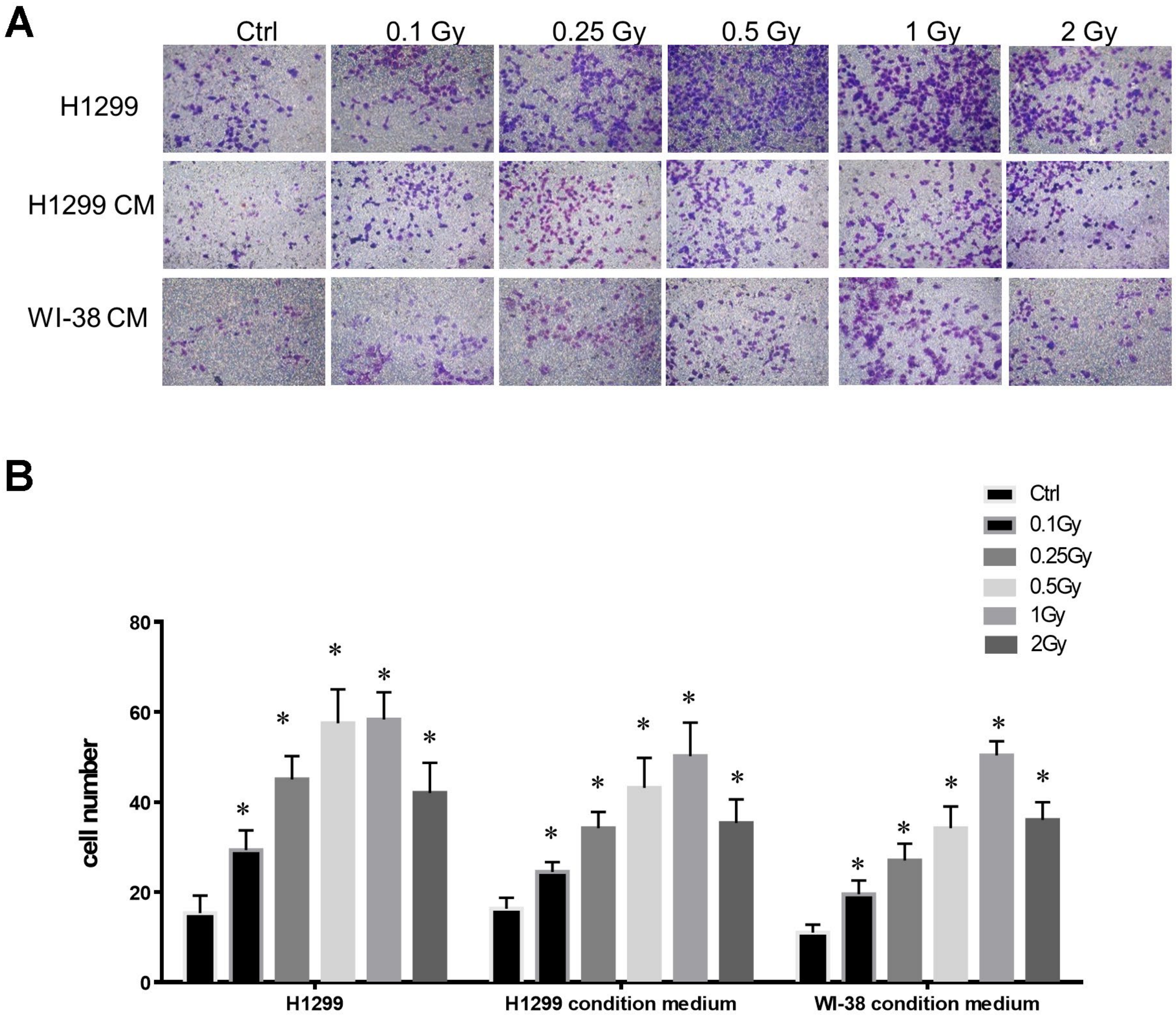

Supplementary Figure 1. *In vitro* invasion assay. (**A**) H1299 cells were either irradiated by different doses of X-rays, or treated with conditioned medium (CM) of irradiated H1299 cells or irradiated WI-38 cells. (**B**) Quantification of the number of cells transversing matrigel coated transwells at different conditions. $^{*}p < 0.05$ compared to ctrl.

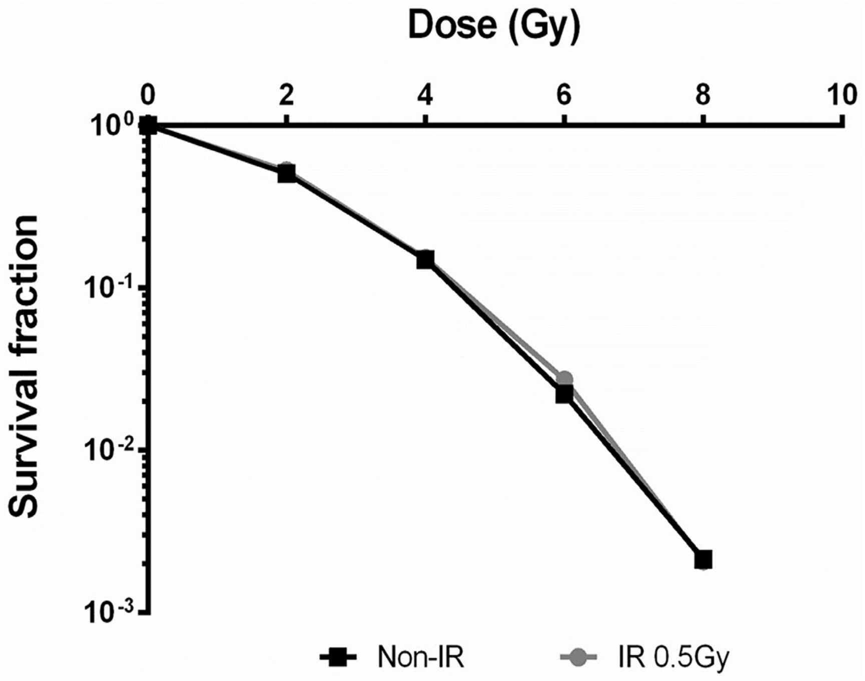

Supplementary Figure 2. Colony formation assay for analysis of survival fractions of LDR (0.5Gy) pretreated H1299 cells or untreated cells exposed to 2 to 8 Gy of X-rays.

A

	a	b	c	d	e	f	g	h	I	j	k	l
1	Pos	Pos	Neg	Neg	ENA-78	GCSF	GM-CSF	GRO	GRO-α	I-309	IL-1α	IL-1 β
2	Pos	Pos	Neg	Neg	ENA-78	GCSF	GM-CSF	GRO	GRO-α	I-309	IL-1α	IL-1 β
3	IL-2	IL-3	IL-4	IL-5	IL-6	IL-7	IL-8	IL-10	IL-12 p40p70	IL-13	IL-15	IFN-γ
4	IL-2	IL-3	IL-4	IL-5	IL-6	IL-7	IL-8	IL-10	IL-12 p40p70	IL-13	IL-15	IFN-γ
5	MCP-1	MCP-2	MCP-3	MCSF	MDC	MIG	MIP-1 δ	RANTES	SCF	SDF-1	TARC	TGF- β1
6	MCP-1	MCP-2	MCP-3	MCSF	MDC	MIG	MIP-1 δ	RANTES	SCF	SDF-1	TARC	TGF- β1
7	TNF-α	TNF-β	EGF	IGF-I	Angiogenin	Oncostatin M	Thrombopoietin	VEGF	PDGF BB	Leptin	Neg	Pos
8	TNF-α	TNF-β	EGF	IGF-I	Angiogenin	Oncostatin M	Thrombopoietin	VEGF	PDGF BB	Leptin	Neg	Pos

Note: GRO detects CXCL1, CXCL2, CXCL3; GRO-α detects only CXCL1
Note: IL-12 p40p70 detects both IL-12 p40 and IL-12 p70
Note: VEGF detects VEGF-165 and VEGF-121

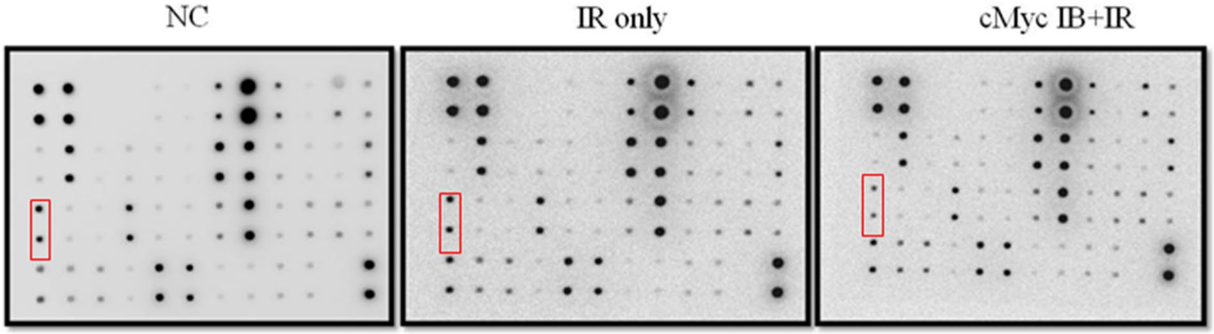

B

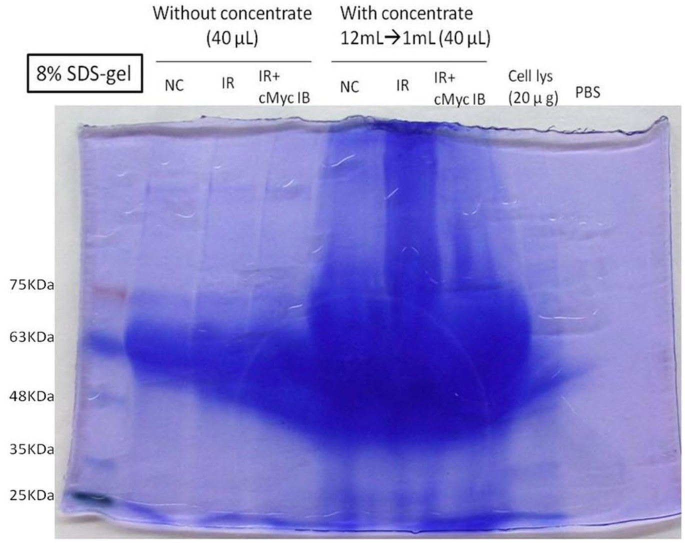

Supplementary Figure 3. Cytokine array analysis of irradiated CM. (**A**) Cytokine array was used to detect the cytokines released in CM (0.5Gy irradiated) with or without c-Myc inhibitor (Myc IB, 50 µm) treatment. (**B**) SDS-PAGE with Coomassie blue staining of ICM with or without spin concentration.

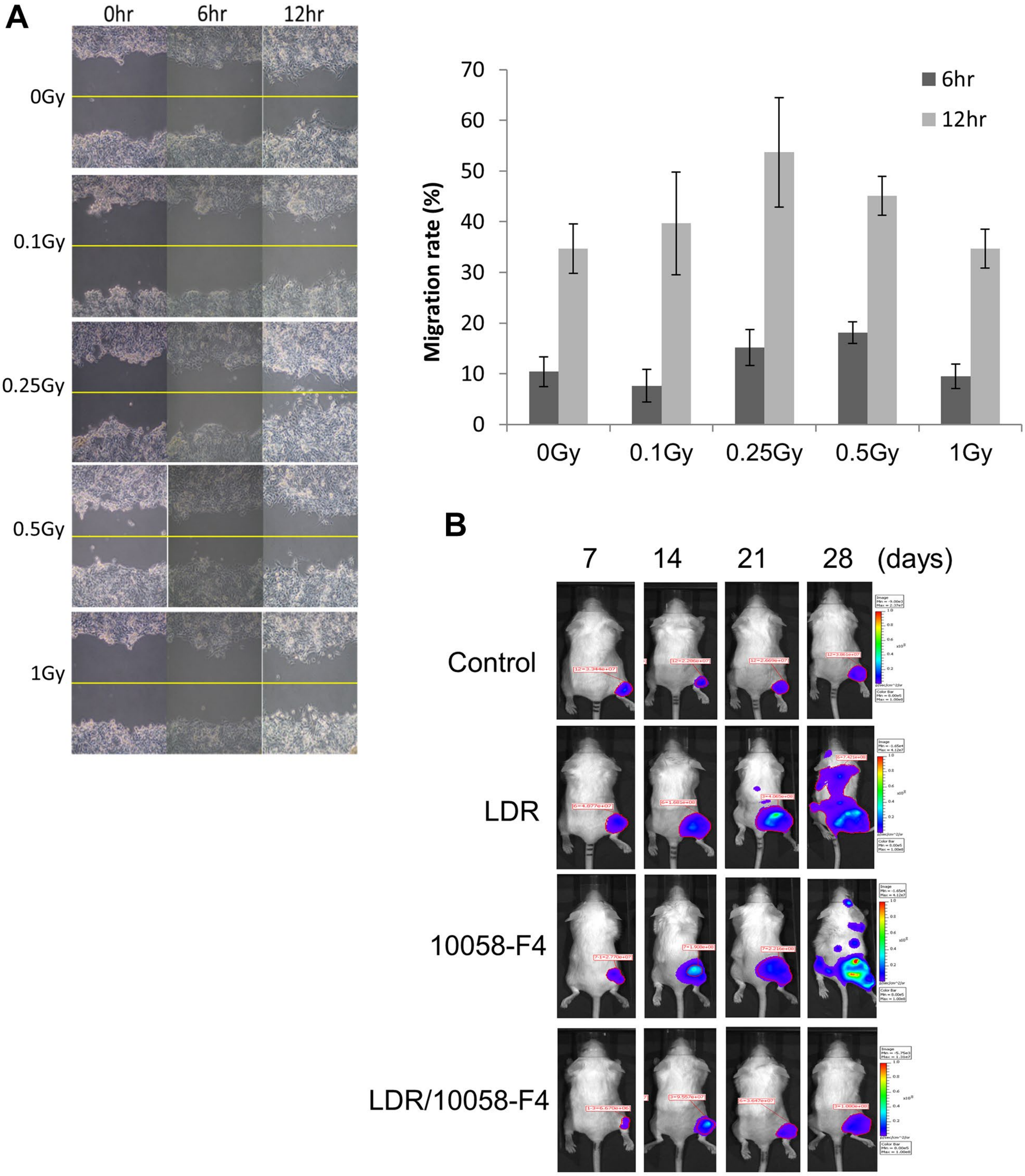

Supplementary Figure 4. Effects of LDR on 4T1-3R murine breast cancer generated syngeneic tumor model. (**A**) Wound healing assay for analysis of 4T1-3R cell migration after exposure to LDR. (**B**) Time course dependent *in vivo* bioluminescent imaging for detecting the tumor metastasis after LDR and/or 10058-F4 (25 mg/kg) treatment in the syngeneic tumor model.

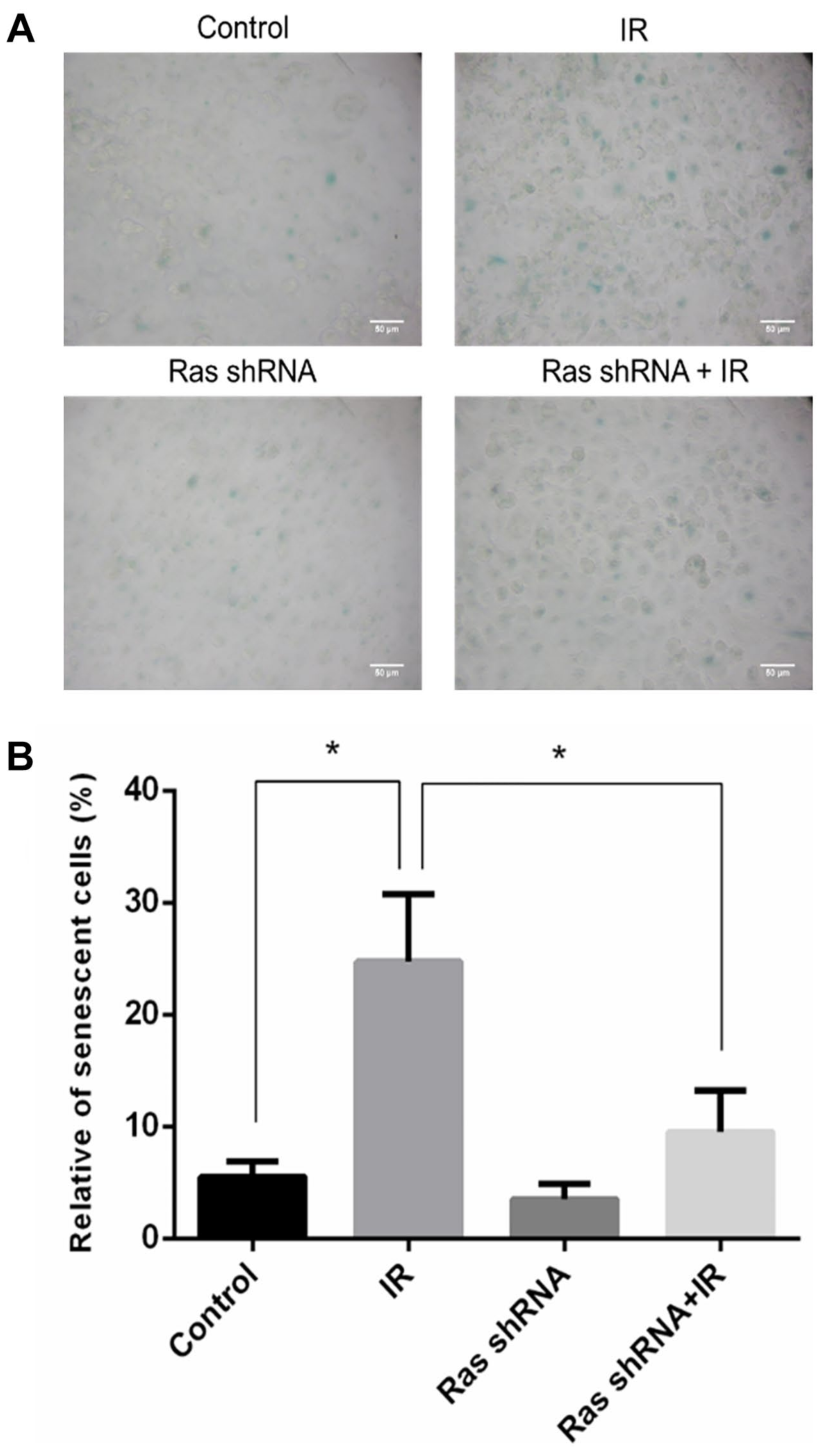

Supplementary Figure 5. Knockdown of K-Ras suppressed LDR induced cell senescence. (**A**) SA-β-gal staining in H1299 cells treated with Ras shRNA and/or 0.5Gy of X-rays. (**B**) Quantification of SA-β-gal staining. $^{*}p < 0.05$.
