# Supplementary material for: Involvement of c-Myc in low dose radiation-induced senescence enhanced migration and invasion of unirradiated cancer cells
Source: Aging (Albany NY). 2021 Sep 22;13(18):22208–31. doi: 10.18632/aging.203527 (PMC8507273; doi:10.18632/aging.203527)
Supplement: Supplementary Table 1 [file aging-13-203527-s002.pdf]

SUPPLEMENTARY TABLE

Supplementary Table 1. Densitometric measurement of human cytokine array blots[a].

Cytokine type	IR only	Myc IB + IR	Cytokine type	IR only	Myc IB + IR	Cytokine type	IR only	Myc IB + IR
	0.89	1.30		0.84	1.13		1.20	1.33
ENA-78	0.58	0.89	IL-8	0.75	1.05	SCF	0.79	1.41
	1.17	2.09		0.98	1.13		0.62	0.87
GCSF	1.17	1.74	IL-10	0.93	1.05	SDF-1	0.67	1.07
	2.22	2.22		1.02	1.11		0.66	0.98
GM-CSF	2.52	2.52	IL-12p40p70	1.32	1.33	TARC	0.74	1.11
	1.13	1.38		1.02	0.89		0.67	1.00
GRO	1.01	1.23	IL-13	1.03	1.27	TGF-β1	0.93	1.04
	2.63	2.44		0.79	0.86		1.44	1.17
GRO-α	2.80	3.21	IL-15	0.63	0.84	TNF-α	2.23	1.95
	1.58	3.01		0.81	0.73		0.83	0.70
I-309	2.21	4.07	IFN-γ	0.58	0.82	TNF-β	1.36	1.26
	0.59	0.83		1.19	0.85		0.91	0.66
IL-1α	0.94	1.22	MCP-1	1.54	0.95	EGF	1.14	0.89
	0.59	0.79		1.90	1.38		1.00	1.22
IL1β	0.81	1.02	MCP-2	0.96	0.65	IGF-I	0.97	1.04
	2.11	2.54		0.99	0.75		0.70	0.59
IL-2	2.07	1.79	MCP-3	0.83	0.83	Angiogenin	0.80	0.79
	1.18	1.73		0.68	0.77		0.92	0.86
IL-3	1.33	1.31	MCSF	0.61	0.60	Oncostatin M	0.73	0.84
	1.14	1.57		0.56	0.76		0.93	1.37
IL-4	1.59	1.66	MDC	0.56	0.69	Thrombopietion	0.83	0.90
	0.75	1.11		0.54	1.01		0.65	1.79
IL-5	0.61	1.01	MIG	0.58	0.75	VEGF	0.66	0.96
	0.69	0.79		0.75	1.06		0.55	0.81
IL-6	0.77	0.76	MIP-1δ	0.74	1.00	PDGF BB	0.69	0.95
	0.66	0.86		0.62	0.76		0.64	1.24
IL-7	0.57	1.10	RANTES	0.44	0.64	Leptin	0.82	1.12

[a]Each datum has been normalized to the results of unirradiated cells.
